# Encapsulation of Metal Nanoparticles by Metal–Organic Framework Imaged with In Situ Liquid Phase Transmission Electron Microscopy

**DOI:** 10.1002/advs.202500984

**Published:** 2025-04-17

**Authors:** Guoming Lin, Utkur Mirsaidov

**Affiliations:** ^1^ Department of Physics National University of Singapore Singapore 117551 Singapore; ^2^ Centre for BioImaging Sciences Department of Biological Sciences National University of Singapore Singapore 117557 Singapore; ^3^ Centre for Advanced 2D Materials and Graphene Research Centre National University of Singapore Singapore 117546 Singapore; ^4^ Department of Materials Science and Engineering National University of Singapore Singapore 117575 Singapore

**Keywords:** in situ TEM, metal–organic framework, nanoparticles

## Abstract

Metal nanoparticle@metal−organic framework (NP@MOF) composites hold promise for potential applications in gas storage, catalysis, sensing, environmental monitoring, and biomedicine. Despite their importance, details of how MOFs encapsulate the NPs to form NP@MOF hybrid nanostructures are largely unexplored. Here, using ultra‐low electron‐flux in situ liquid phase transmission electron microscopy (LP‐TEM), the encapsulation of Au NPs with zeolitic imidazolate framework‐8 (ZIF‐8) is visualized. These observations reveal that the speeds at which MOFs nucleate on the NP's surface impact the shell's shape. At low concentrations of MOF precursor, NPs are encapsulated with well‐defined single‐crystalline MOF shells, while at high concentrations, MOFs tend to nucleate and grow from multiple sites on the NP surface, resulting in irregularly shaped polycrystalline MOF shells. This approach, which uses a very low electron flux to image the synthesis of Au@ZIF‐8 nanostructures, can be extended to imaging crucial processes in many other beam‐sensitive materials and help design hybrid systems for a broad range of applications.

## Graphical abstract

In situ liquid‐phase transmission electron microscopy (LP‐TEM) study reveals the pathways through which metal‐organic frameworks (MOFs) encapsulate metal nanoparticles (NPs) and explains why some MOF shells of NP&MOF nanostructures are uniform and single crystalline, while others are nonuniform and polycrystalline.

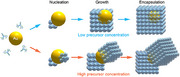

## 1 Introduction

Metal–organic frameworks (MOFs) are a class of highly porous materials with significant potential for applications in gas storage, separation, and catalysis.^[^ ^1^ ^]^ The encapsulation of functional nanoparticles (NPs) within MOFs can produce hybrid systems, allowing for improved control over NP functionality and thereby enhancing their performance for a broad range of applications.^[^ ^2^, ^3^, ^4^, ^5^, ^6^ ^]^ For instance, metal NPs, commonly used as catalysts in various heterogeneous catalytic reactions, often suffer from deactivation due to aggregation and sintering.^[^ ^7^, ^8^, ^9^ ^]^ Encapsulating these metal NPs within MOFs not only stabilizes them but also markedly increases their catalytic activity by concentrating reactant molecules on the NP surfaces. Additionally, the tunable pore structure of MOFs enables precise adjustments of catalytic selectivity, helping to minimize side reactions and enhancing the yield of target products.^[^ ^10^, ^11^, ^12^, ^13^ ^]^ There are two approaches to synthesizing NP@MOF structures. The first approach is to grow MOF directly on the NPs,^[^ ^14^ ^]^ and the second one is to load the MOFs with metal precursor, followed by chemical reduction of the metal ions within the MOF.^[^ ^15^ ^]^ Since the chemical reduction step in the second approach often requires exposing MOFs to prolonged elevated temperatures, it causes damage to their crystal structure. Hence, for applications requiring a well‐defined porous structure of MOFs, the first approach is more desirable.

Despite the importance of these hybrid systems, the exact mechanisms by which MOFs grow to encapsulate NPs remain unknown. This gap in understanding arises from the limitations of current in situ methods, such as X‐ray diffraction,^[^ ^16^, ^17^ ^]^ and small‐angle and wide‐angle X‐ray scattering,^[^ ^18^ ^]^ which lack the resolution needed to track individual encapsulation events and elucidate the pathways through which these hybrid NP@MOF systems form.

Imaging the real‐time encapsulation of individual NPs by a MOF shell using in situ liquid‐phase transmission electron microscopy (LP‐TEM) can overcome this challenge and provide crucial insights into the encapsulation process.^[^ ^19^, ^20^, ^21^, ^22^, ^23^ ^]^ While this imaging technique can be readily applied to purely metallic systems, such as Au@Pd,^[^ ^24^ ^]^ Au@Ag,^[^ ^25^ ^]^ and Au@Cu_2_O,^[^ ^26^ ^]^ imaging dynamic processes involving MOFs is more challenging. This difficulty arises because MOFs are highly sensitive to electron beams and are easily damaged during imaging.^[^ ^27^, ^28^ ^]^ This issue can be mitigated by employing the ultra‐low electron flux in situ TEM imaging approach that we have previously developed.^[^ ^21^ ^]^

## 2 Results and Discussion

Here, we use in situ LP‐TEM to investigate the encapsulation of individual Au NPs with a zeolitic imidazolate framework‐8 (ZIF‐8) shell. We chose ZIF‐8 as the encapsulating MOF because it is a well‐characterized MOF system. The inherent beam sensitivity of MOFs, which forces us to capture dynamic processes with ultra‐low electron flux, makes high‐resolution imaging of the encapsulation process challenging. High resolution is necessary to confirm that the MOFs are crystalline and remain undamaged by the ionizing electron beam used for imaging. To address this, we add cetyltrimethylammonium chloride (CTAC) into the precursor solution as a surfactant,^[^ ^29^ ^]^ facilitating the formation of cuboidal MOF shells (Figures S1 and S2, Supporting Information). This cuboidal morphology does not depend on the shape or orientation of the encapsulated Au NPs and serves as a clear indicator of the MOFs' being crystalline, indicating that they are not damaged by the electron beam.^[^ ^21^ ^]^

**Figure 1:**
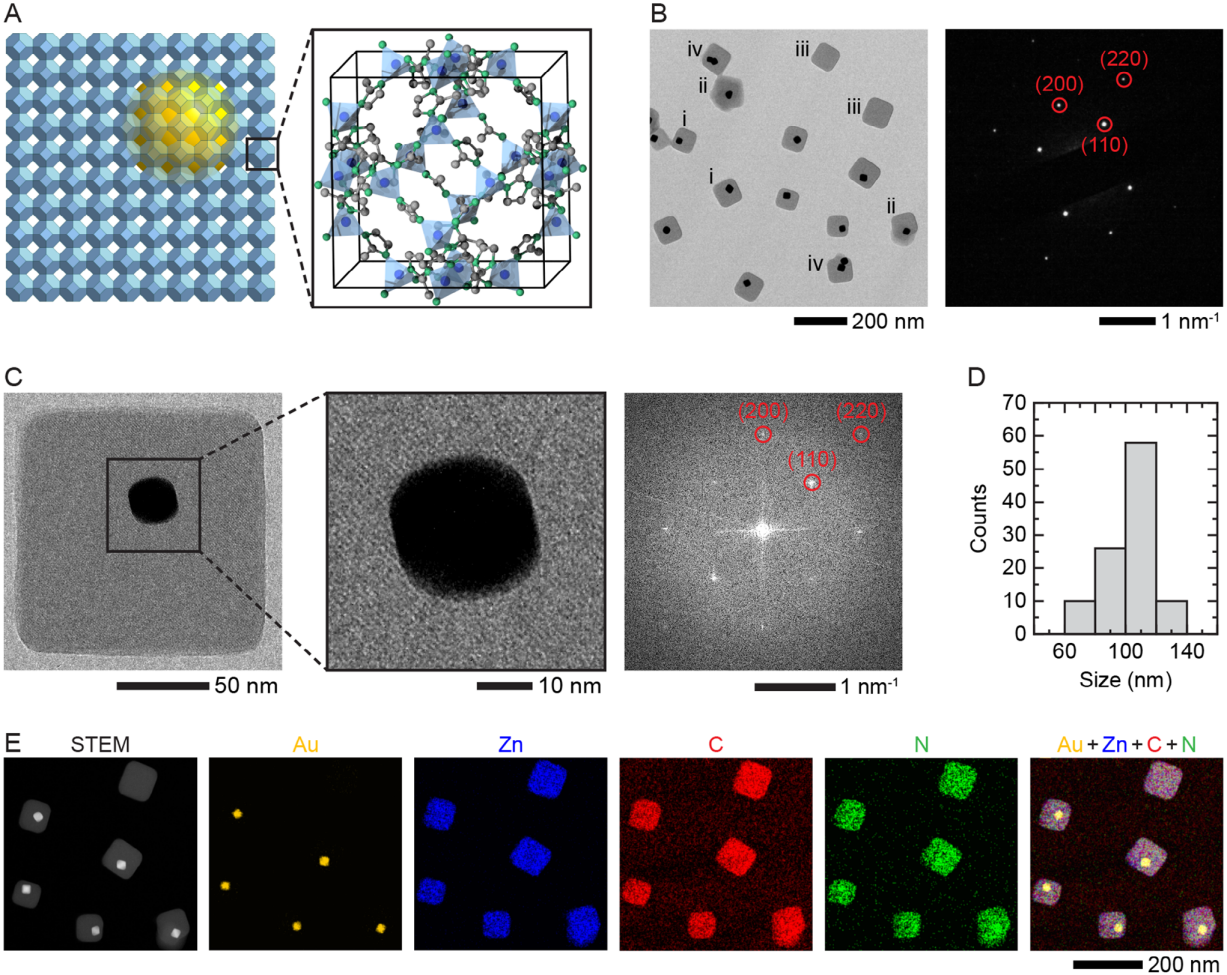
Core−shell Au@ZIF‐8 nanostructures. A) Schematic of a Au@ZIF‐8 nanostructure viewed along the [100] orientation of ZIF‐8, and corresponding unit cell of ZIF‐8. B) TEM and electron diffraction images of Au@ZIF‐8 nanostructures. Red circles identify (110), (220), and (200) diffraction spots of ZIF‐8. C) TEM images and corresponding Fourier transform pattern of an individual Au@ZIF‐8 nanostructure. D) Size distribution of Au@ZIF‐8 nanostructures. E) STEM and corresponding EDX images of Au@ZIF‐8 nanostructures showing their elemental composition.

**Figure** **1**A–D displays schematic and TEM images of Au NPs encapsulated by cuboidal ZIF‐8 shells and their size distribution, while Figure 1E shows energy‐dispersive X‐ray spectroscopy (EDX) maps detailing the composition of these Au@ZIF‐8 nanostructures. In solutions without Au NPs, only cuboidal ZIF‐8 NPs form as expected (Figure S1, Supporting Information). Notably, while most individual Au NPs are encapsulated by single cuboidal ZIF‐8 shells (Figure 1B‐i), there are some exceptions. For instance, we occasionally found NPs encapsulated with multifaceted shells (Figure 1B‐ii), presumably resulting from the simultaneous nucleation of two or more cuboidal MOF shells around a single NP. In other cases, MOF nanocubes may nucleate independently without encapsulating any NPs (Figure 1B‐iii), or a MOF cube may form around two attached NPs (Figure 1B‐iv).

**Figure 2:**
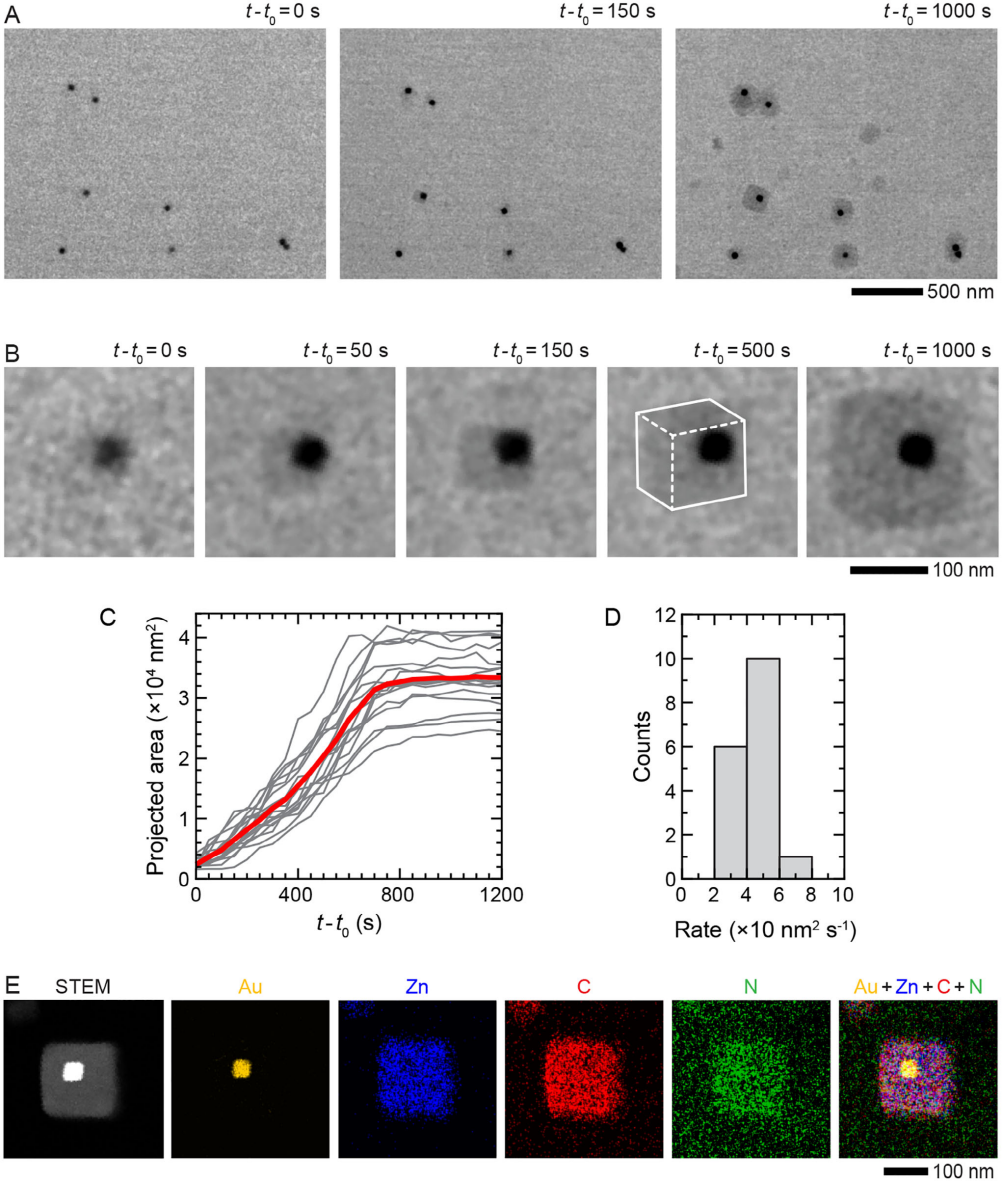
Formation of core−shell Au@ZIF‐8 nanostructures. A) In situ TEM image series showing the encapsulation of Au NPs by ZIF‐8 shells in an aqueous solution comprising 0.4 m 2‐MeIm, 6 mm Zn(NO_3_)_2_, and 0.25 mm CTAC in a liquid cell at room temperature (Video S4, Supporting Information). Here, *t*_0_ represents the time point when the imaging started. B) Enlarged view of the TEM image series shown in (A) depicting the details of Au@ZIF‐8 nanostructure formation. C) Plot of projected areas of Au@ZIF‐8 nanostructures as a function of time. D) Corresponding histogram depicting the distribution of growth rates obtained from plots in (C). E) STEM and corresponding EDX images of a Au@ZIF‐8 nanostructure on the SiN_x_ window of a liquid cell after the in situ growth.

In order to understand how the core–shell nanostructures form, we track their evolution with in situ LP‐TEM using ultra‐low electron flux of ∼0.5 e^−^ nm^−2^ s^−1^ to minimize the effect of an electron beam on the overgrowth process (Figure S3, Videos S1–S3, Supporting Information).^[^ ^21^ ^]^ In situ TEM image series in **Figure** **2**A,B shows the encapsulation process of uniformly dispersed Au NPs that have been introduced into a liquid flow cell together with the ZIF‐8 precursor. As the solution (comprising 0.4 m 2‐MeIm and 6 mM Zn(NO_3_)_2_, 0.25 mM CTAC, and Au NPs) reaches the window of the liquid cell, a shell starts to grow on the NP, as evidenced by a low contrast layer forming around the NPs (Figure 2B: *t* −  *t*_0_ =  50 s). Within ≈10^3^ s, the growth ceases, forming a mature cuboidal MOF shell with a side length of ≈150 nm surrounding the Au NP (Figure 2B: *t* −  *t*_0_ =  1000 s). In our experiments, more than 85% of the individual NPs or NP aggregates get encapsulated, indicating that they serve as sites for heterogeneous nucleation of MOFs. Note that NPs are mostly encapsulated by single well‐defined cuboidal MOF shells, as confirmed by scanning TEM (STEM) and EDX images shown in Figure 2E, indicating that shells grow from a single nucleus on the surface of the NPs. This will become important later when we contrast this growth to the growth of the shells from a precursor solution with higher concentrations. A comparison of the growth trajectories of individual Au@ZIF‐8 nanostructures shown in Figure 2A and Figure S4 (Supporting Information) reveals that the projected area of a MOF shell grows at a rate of ≈40 nm^2^ s^−1^ (Figure 2C,D). After the in situ growth, we disassembled the liquid cell to perform EDX analysis on the in situ grown Au@ZIF‐8 nanostructures directly on top of the SiN_x_ window of the liquid cell, which confirmed the encapsulation of Au NPs by ZIF‐8 (Figure 2E).

**Figure 3:**
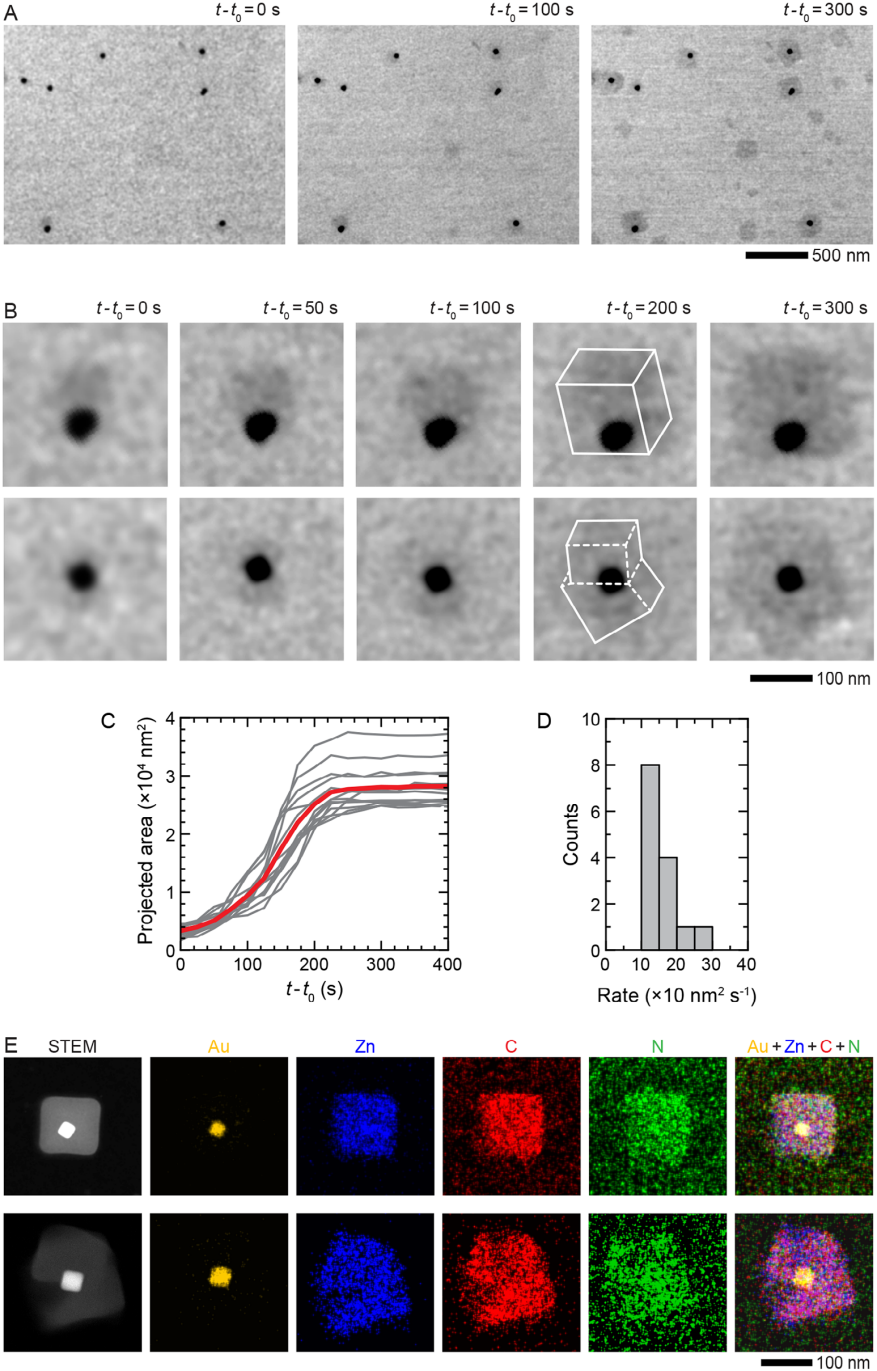
Formation of core−shell Au@ZIF‐8 nanostructures in a high‐concentration ZIF‐8 precursor solution. A) In situ TEM image series showing the encapsulation of Au NPs by single or multiple ZIF‐8 shells in an aqueous solution comprising 0.6 m 2‐MeIm, 9 mm Zn(NO_3_)_2_, and 0.25 mm CTAC in a liquid cell at room temperature (Video S5, Supporting Information). Here, *t*_0_ represents the time point when the imaging started. B) Enlarged views of the TEM image series shown in (A) depicting the details of two Au@ZIF‐8 nanostructures formation. C) Plot of projected areas of Au@ZIF‐8 nanostructures as a function of time. D) Corresponding histogram depicting the distribution of growth rates obtained from plots in (C). E) STEM and corresponding EDX images of a Au@ZIF‐8 nanostructure on the SiN_x_ window of a liquid cell after the in situ growth.

To tease out the details of the encapsulation process, we performed a similar in situ study at a higher precursor concentration (0.6 m 2‐MeIm and 9 mm Zn(NO_3_)_2_) while keeping the concentration of Au NPs and CTAC the same (**Figure** **3A,B**; Figure S5, Supporting Information). As shown in Figure 3C,D, ZIF‐8 shells grow three times as fast as in the lower concentration precursor solution described in Figure 2C,D (i.e., the average growth rate of ≈160 vs ≈40 nm^2^ s^−1^). While a faster growth rate with increasing precursor concentration is expected, the observed variation in the morphology of the ZIF‐8 shells was somewhat surprising. We found that in addition to Au@ZIF‐8 nanostructures with regular cuboidal shells, there were many nanostructures with irregularly shaped, multifaceted shells. A closer examination and comparison of the growth process and final products reveal that in contrast to regular cuboidal shells (Figure 3B,E: *top panels*), these shells comprise two or three MOF nanocubes that nucleate early on and grow on the surface of a Au NP (Figure 3B,E: *bottom panels*). Hence, the shell of a Au@ZIF‐8 nanostructure is no longer single crystalline but consists of two or more ZIF‐8 nanocubes (Figure S6, Supporting Information).

**Figure 4:**
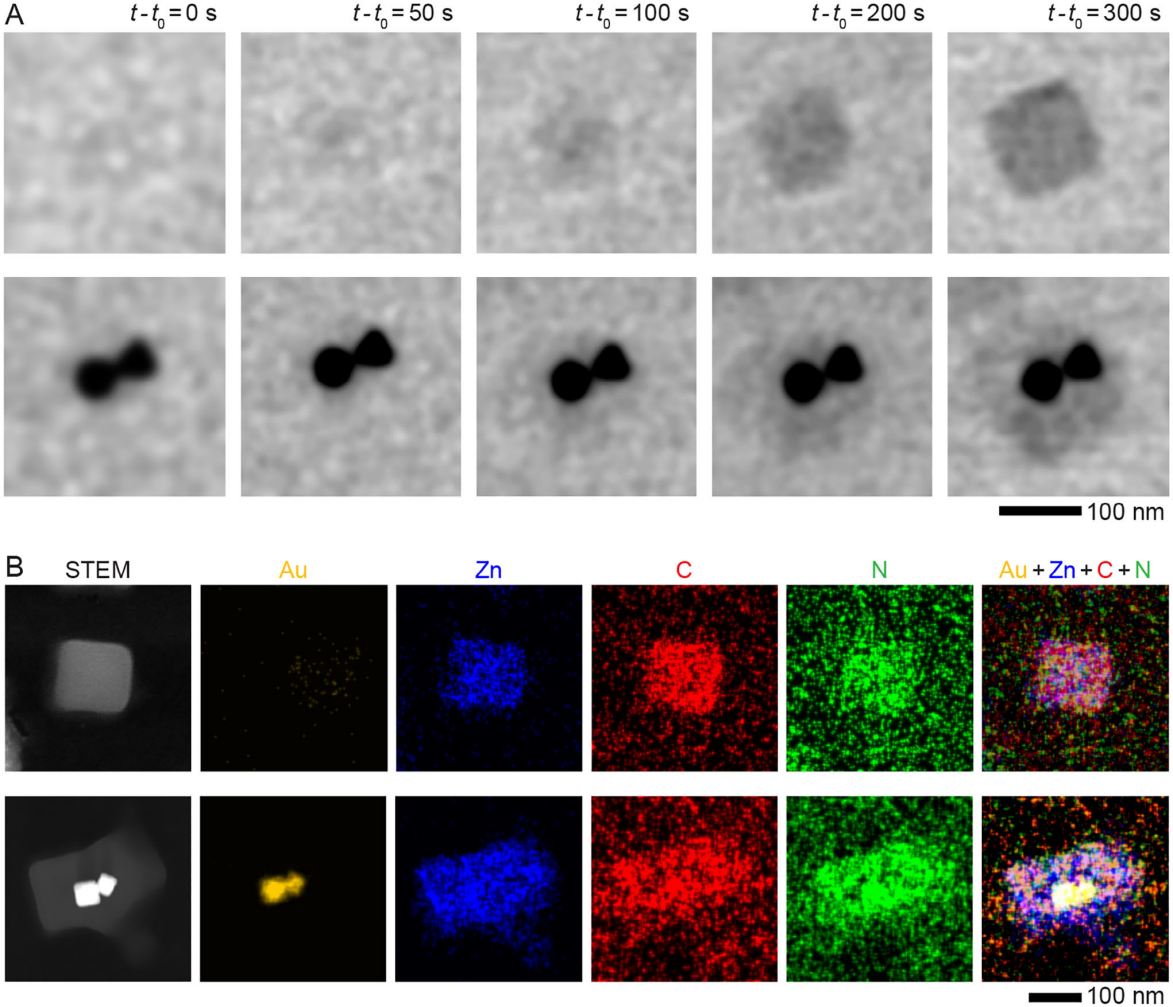
Encapsulation process of Au NPs at high‐concentration of ZIF‐8 precursor solution. A) In situ TEM image series showing the growth of an empty cuboidal ZIF‐8 NP (*bottom panel*) and the encapsulation of two Au NPs by multiple ZIF‐8 shells and (*top panel*) in an aqueous solution comprising 0.6 m 2‐MeIm, 9 mm Zn(NO_3_)_2_, and 0.25 mm CTAC in a liquid cell at room temperature (Video S5, Supporting Information) showing the encapsulation process of two Au NPs by ZIF‐8 NPs, and growth process of empty ZIF‐8 NP. B) STEM and corresponding EDX images of a Au@ZIF‐8 nanostructure and an empty ZIF‐8 NP on the SiN_x_ window of a liquid cell after the in situ growth.

What is also consistent with growth at a high precursor concentration is that many more NPs are encapsulated with ZIF‐8 shells than at lower concentrations described in Figure 2 (97% vs 85%). However, with this improvement in the encapsulation rate, the yield of hybrid NPs drops, meaning that more and more ZIF‐8 nanocubes nucleate spontaneously in the solution without encapsulating the Au NPs (**Figure** **4A,B**: *top panels*). For example, at lower concentration (0.4 m 2‐MeIm and 6 mm Zn(NO_3_)_2_) described in Figure 2, the ratio of Au@ZIF‐8 nanostructures to empty ZIF‐8 NPs is ≈2:3, while this ratio drops to ≈1:4 at higher concentration (0.6 m 2‐MeIm and 9 mm Zn(NO_3_)_2_), as can be seen from many more empty ZIF‐8 NPs in Figure 3A. Moreover, similar to the nanostructures shown in Figure 1B‐iv, we also find that two attached Au NPs are encapsulated in two ZIF‐8 shells (Figure 4A,B: *bottom panels*). Similar nanostructures can also be seen during low‐concentration synthesis, with two attached Au NPs serving as two nucleation sites (Figure S4B, Supporting Information). Here, it is worth noting that the formation of ZIF‐8 is a diffusion‐limited reaction because, while increasing the concentration of ZIF‐8 precursor increases the nucleation rate, the temperature appears to have very little impact on the nucleation process (Figure S7, Supporting Information).

## 3 Conclusion

**Figure 5:**
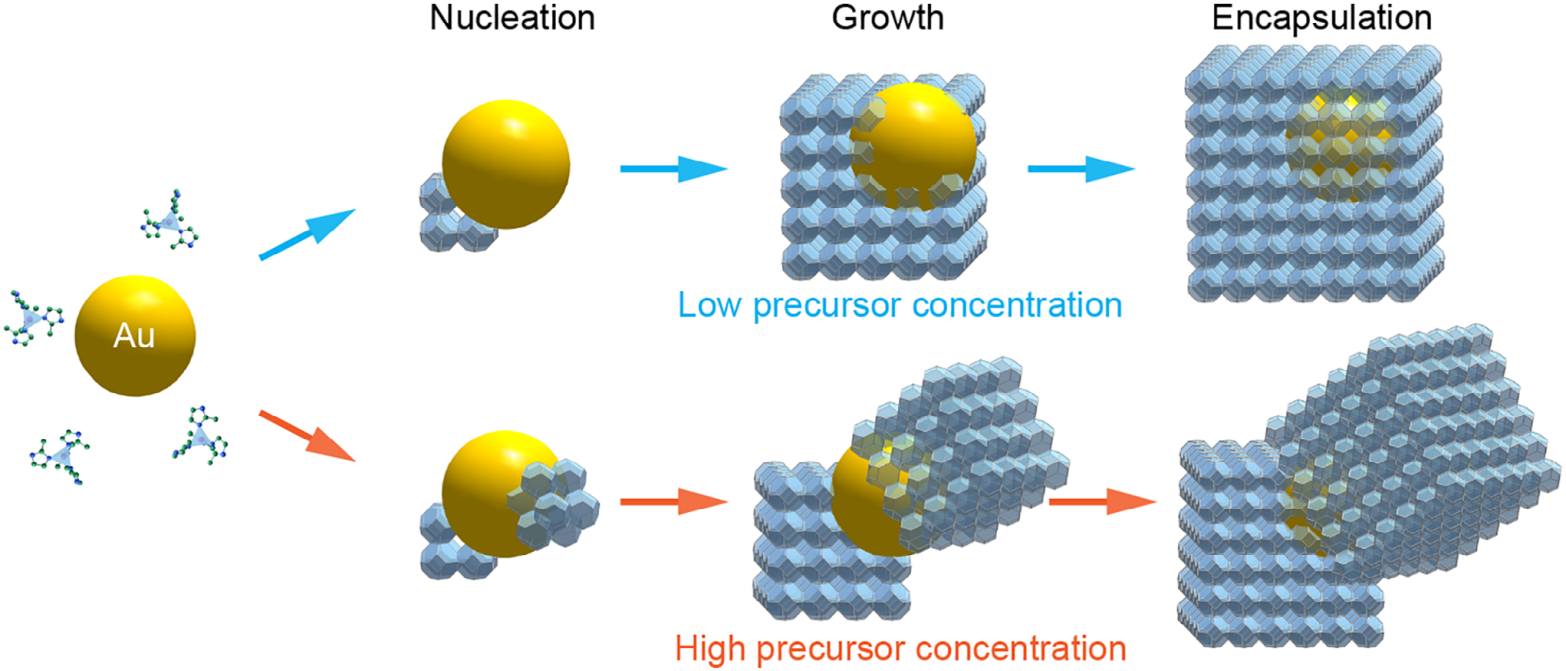
Pathway of formation of Au@ZIF‐8 nanostructures. Schematic of the proposed pathway describing the growth of a MOF shell on a Au NP. At higher precursor concentrations, MOF can nucleate at multiple sites on the surface of the NP, resulting in the NP being encapsulated by more than one cuboidal MOF shell.

Controlling the shape uniformity and crystallinity of the MOF shells is important in synthesizing reliable NP@MOF nanostructures for applications in catalysis. Our in situ observations tracking the nanoscopic details of the encapsulation of metallic NPs reveal that an overgrowth of a uniform MOF shell results from heterogeneous nucleation of MOF on the NP surface and its subsequent growth (**Figure** **5**). Increasing the probability of multiple nucleation events on the NP by increasing the concentration of MOF precursor produces nonuniform, polycrystalline shells. Hence, insights from this and other future studies focusing on the growth mechanisms of MOFs by direct imaging can aid in developing and optimizing the synthesis of hybrid organic–inorganic nanostructures for various chemical and biomedical applications.

## 4 Experimental Section

### Materials

The following reagents (from Sigma–Aldrich Co., St. Louis, MO, USA) were used in our study: zinc nitrate hexahydrate ((Zn(NO_3_)_2_·6H_2_O, Cat. No. 228 737), 2‐methylimidazole (2‐MeIm, Cat. No. M50850), cetyltrimethylammonium bromide (CTAB, Cat. No. H9151), cetyltrimethylammonium chloride (CTAC, 25 wt.% in H_2_O, Cat. No. 292 737), gold (III) chloride trihydrate (HAuCl_4_·3H_2_O, Cat. No. 254 169), and trisodium citrate dihydrate (Cat. No. S1804). All aqueous solutions were prepared using deionized water with a resistivity of 18.2 MΩ cm.

### Synthesis of Au Octahedron NPs

The octahedron Au NPs were synthesized by slightly modifying the protocol described by Chang et al.^[^ ^30^ ^]^ A 1.25 mL of 10 mm HAuCl_4_ solution and 0.25 mL of 100 mm trisodium citrate solution were mixed with 50 mL of 100 mm CTAB solution. This mixture was then loaded into a 100‐mL pressure vessel and heated at 120^ °^C for 6 h to form the Au NPs. These NPs were washed twice in DI water and then in 5 mm CTAC solution by centrifugation at 4600 g for 8 min. The washed NPs were then dispersed in 2.5 mL of 5 mm CTAC solution for 24 h to allow CTAB‐to‐CTAC ligand exchange. Prior to the synthesis of Au@ZIF‐8 nanostructures, 0.2 mL of these Au‐CTAC NPs suspension was thoroughly mixed with 1.8 mL DI water and then centrifuged at 3400 g for 5 min. Then, the supernatant was removed, leaving behind 0.2 mL of 5 mm Au‐CTAC NPs in a 0.5 mm CTAC solution, which was used for *in‐flask* or in situ synthesis of Au@ZIF‐8 nanostructures.

### Synthesis of Au@ZIF‐8 Nanostructures

The reaction that describes the synthesis of ZIF‐8 can be written as Zn(NO_3_)_2_ · 6H_2_O + C_4_H_6_N_2_ → Zn(C_4_H_5_N_2_)_2_ + H^+^  + NO_3_^−^  + H_2_O.^[^ ^31^ ^]^ Cuboidal Au@ZIF‐8 nanostructures were synthesized by using a modified protocol described by Hu et al.^[^ ^32^ ^]^ First, 0.5 mL of 0.5 mm aqueous CTAC solution and 0.5 mL of 2.4 m 2‐MeIm aqueous solution were mixed and stirred at 500 rpm. After 3 min stirring, 0.5 mL of 36 mm Zn(NO_3_)_2_ aqueous solution was added into this mixture (while stirring) to finalize the preparation of ZIF‐8 precursor solution. After 10 s, 0.5 mL of Au NPs in 0.5 mm CTAC solution was injected into the ZIF‐8 precursor solution. This solution comprising ZIF‐8 precursor solution and the NPs was either introduced into liquid cell for in situ TEM imaging of the Au@ZIF‐8 formation process or stirred for an additional 30 s at 500 rpm and then left undisturbed at room temperature for 1 h for *in‐flask* synthesis of Au@ZIF‐8 nanostructures.

### In Situ LP‐TEM Experiments

In situ studies were conducted inside a liquid cell comprising two (i.e., top and bottom) custom‐fabricated chips, each with a 25‐nm‐thick SiN_x_ window. The windows of the chips were treated with gentle air plasma for 2 min to render their surface hydrophilic and facilitate the flow of precursor. The top and bottom chips were assembled and sealed within a liquid flow holder (Hummingbird Scientific, Lacey, WA, USA), which was connected to ≈25‐cm‐long and 200‐µm‐diameter inlet and outlet fluid tubings. After verifying proper window alignment and confirming that there were no leaks in the flow cell, the entire assembly was loaded into a TEM for in situ imaging. Next, a fresh solution comprising MOF precursor and Au NPs was prepared as described above and was then connected to the inlet fluid tubing. Immediately after this, a vacuum was applied through the outlet port to rapidly draw the precursor solution through the inlet into the cell to avoid any potential growth of MOFs on the NPs before they reached the viewing window of the liquid cell. Pulling liquid by lowering the outlet pressure is a much faster process than pushing the liquid through the inlet with the syringe. It enables recording the synthesis process as early as possible.

### Imaging and Characterization

The STEM imaging and EDX analysis were conducted with a 300 kV Titan TEM (Thermo Fisher Scientific Inc., Hillsboro, OR, USA), equipped with a Bruker XFlash 6T|30 EDX spectrometer (Bruker Nano GmbH, Berlin, Germany) and a Gatan K2 IS CMOS camera (Gatan Inc., Pleasanton, CA, USA). The in situ LP‐TEM studies were performed on a 200 kV JEOL 2010F TEM (JEOL Ltd., Tokyo, Japan) equipped with a Gatan OneView camera (Gatan Inc., Pleasanton, CA, USA). The in situ image series were recorded at a rate of two frames per second with an incident electron beam flux of ≈0.5 e^–^ nm^−2^ s^−1^. All image processing algorithms for analyzing the in situ image sequence files were written in Python 3.7.1.^[^ ^33^ ^]^ Initially, raw dm4 format files generated by Gatan OneView camera were converted into 8‐bit PNG image files. Subsequently, a moving average of five consecutive image frames was used to produce initial averaged frames, from which a region of interest (ROI) was selected. The drift in the ROI frames was tracked using a stationary NP as a reference. The drift coordinates were then used to align the original PNG image frames to obtain drift‐corrected image frames. Next, to enhance the signal‐to‐noise ratio of the image series, once more a moving average of five frames to the drift‐corrected frames was applied. Finally, a 3D Gaussian filter with σ_*x*_ = σ_*y*_  =  2 pixels and σ_*z*_ =  4 pixels were applied to the image stack to remove high‐frequency noise. Here, the *z*‐axis represents the time or image frame number. The projected areas of the NPs were determined by counting the pixels occupied by the NPs.

## Conflict of Interest

The authors declare no conflict of interest.

## Supporting information



Supporting Information

Supplemental Video 1

Supplemental Video 2

Supplemental Video 3

Supplemental Video 4

Supplemental Video 5

## Data Availability

The data that support the findings of this study are available from the corresponding author upon reasonable request.
