# Supplementary material for: Encapsulation of Metal Nanoparticles by Metal–Organic Framework Imaged with In Situ Liquid Phase Transmission Electron Microscopy
Source: Adv Sci (Weinh). 2025 Apr 17;12(25):2500984. doi: 10.1002/advs.202500984 (PMC12224958; doi:10.1002/advs.202500984)
Supplement: Supplementary file 1 — Supporting Information [file ADVS-12-2500984-s004.pdf]

ADVANCED SCIENCE
Open Access

Supporting Information

for *Adv. Sci.*, DOI 10.1002/advs.202500984

Encapsulation of Metal Nanoparticles by Metal–Organic Framework Imaged with In Situ Liquid Phase Transmission Electron Microscopy

*Guoming Lin and Utkur Mirsaidov**

Supporting Information

Encapsulation of Metal Nanoparticles by Metal-Organic Framework Imaged with In Situ Liquid Phase Transmission Electron Microscopy

Guoming Lin[1, 2] *and Utkur Mirsaidov*[1, 2, 3, 4]*

1. Department of Physics, National University of Singapore, Singapore, 117551, Singapore
2. Centre for BioImaging Sciences, Department of Biological Sciences, National University of Singapore, Singapore, 117557, Singapore
3. Centre for Advanced 2D Materials and Graphene Research Centre, National University of Singapore, Singapore, 117546, Singapore
4. Department of Materials Science and Engineering, National University of Singapore, Singapore, 117575, Singapore

*Correspondence: mirsaidov@nus.edu.sg

1. Supporting figures

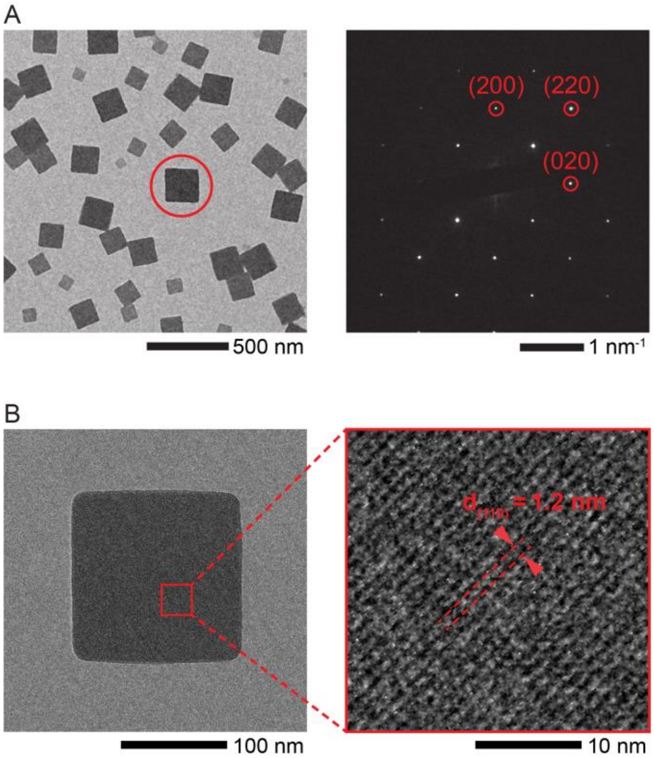

Figure S1. Cuboidal ZIF-8 nanoparticles. (**A**) TEM and electron diffraction images of single-crystalline ZIF-8 nanocubes. (**B**) High-resolution TEM images of a single-crystalline ZIF-8 nanocube.

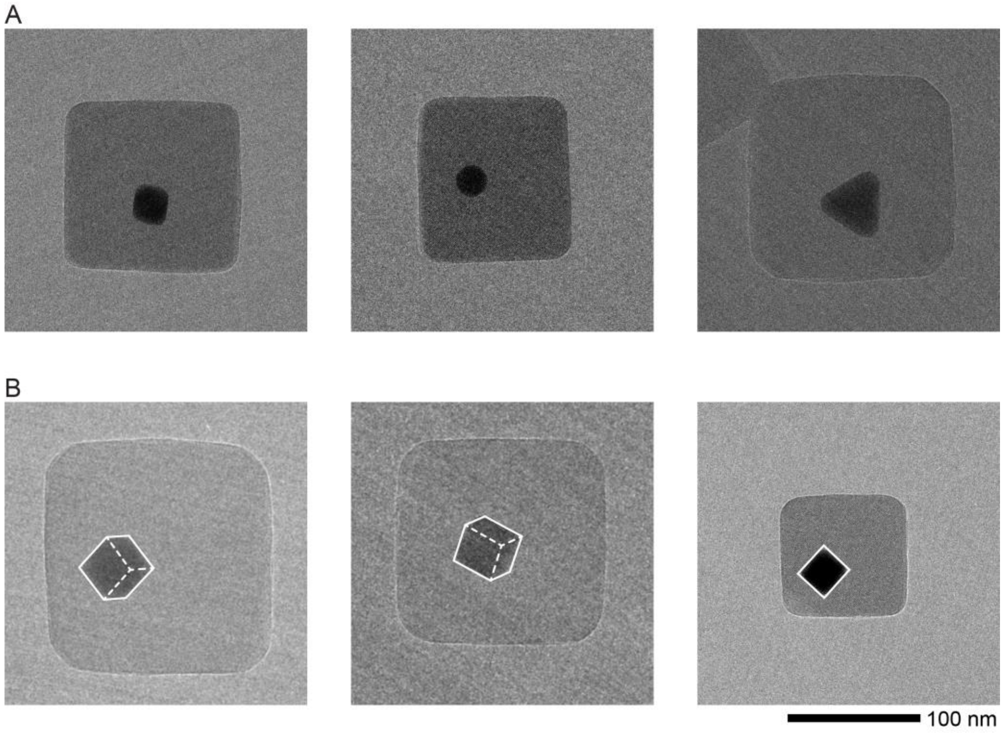

Figure S2. Cuboidal Au@ZIF-8 nanostructures. (**A**) TEM images of octahedral, spherical, and triangular Au NPs with cuboidal ZIF-8 shells. (**B**) TEM images of Au nanocubes with cuboidal ZIF-8 shells.

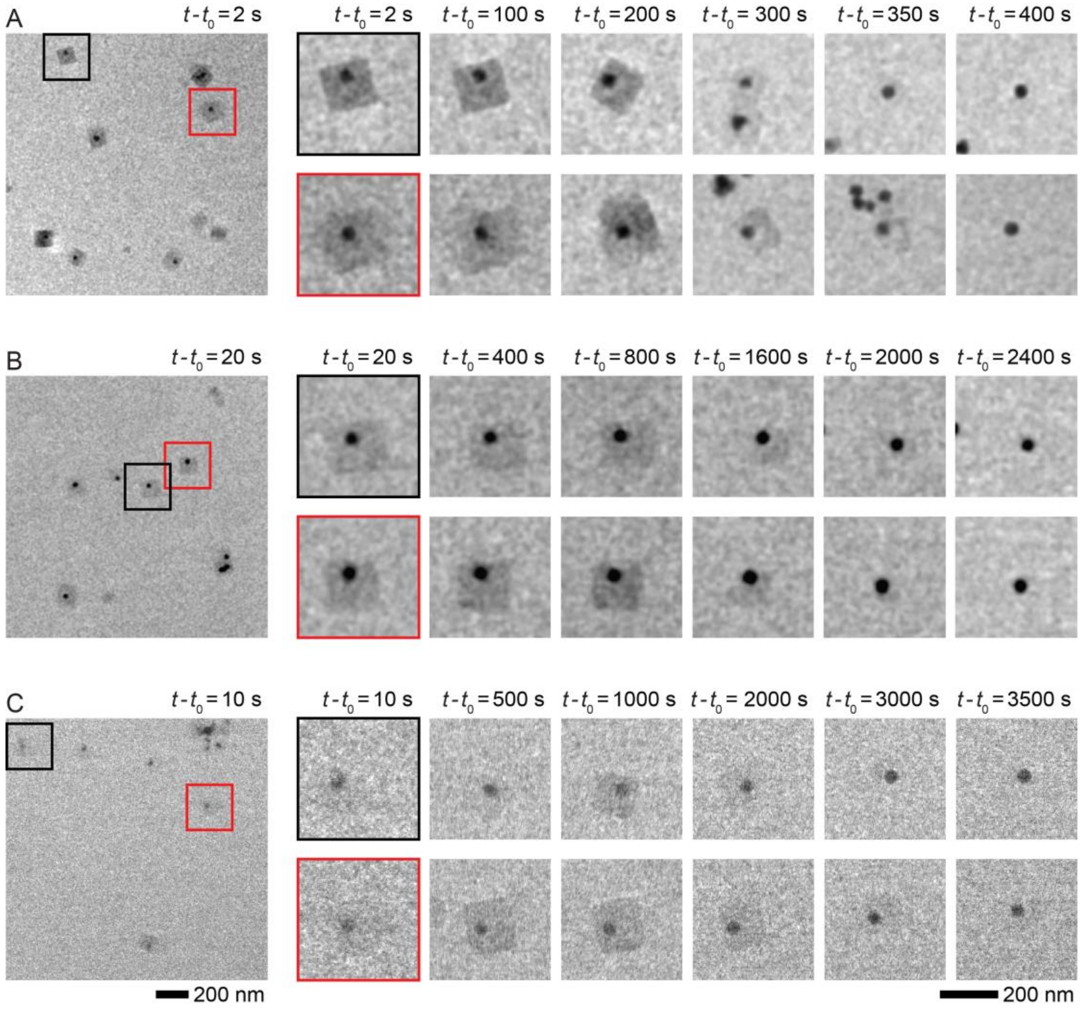

Figure S3. Testing the effect of 200-keV electron beam on the dissolution of ZIF-8 shells. *In situ* TEM image series of pre-synthesized Au@ZIF-8 nanostructures in a precursor solution (0.4 M 2-MeIm + 6 mM $Zn(NO_3)_2$ + 0.25 mM CTAC) imaged using three different electron fluxes: (**A**) ~5 e^{-} nm^{-2} s^{-1} (Supporting Video S1), (**B**) ~0.75 e^{-} nm^{-2} s^{-1} (Supporting Video S2), and (**C**) ~0.5 e^{-} nm^{-2} s^{-1} (Supporting Video S3). Here, shells dissolve at ~350, 2000, and 3500 s, respectively.

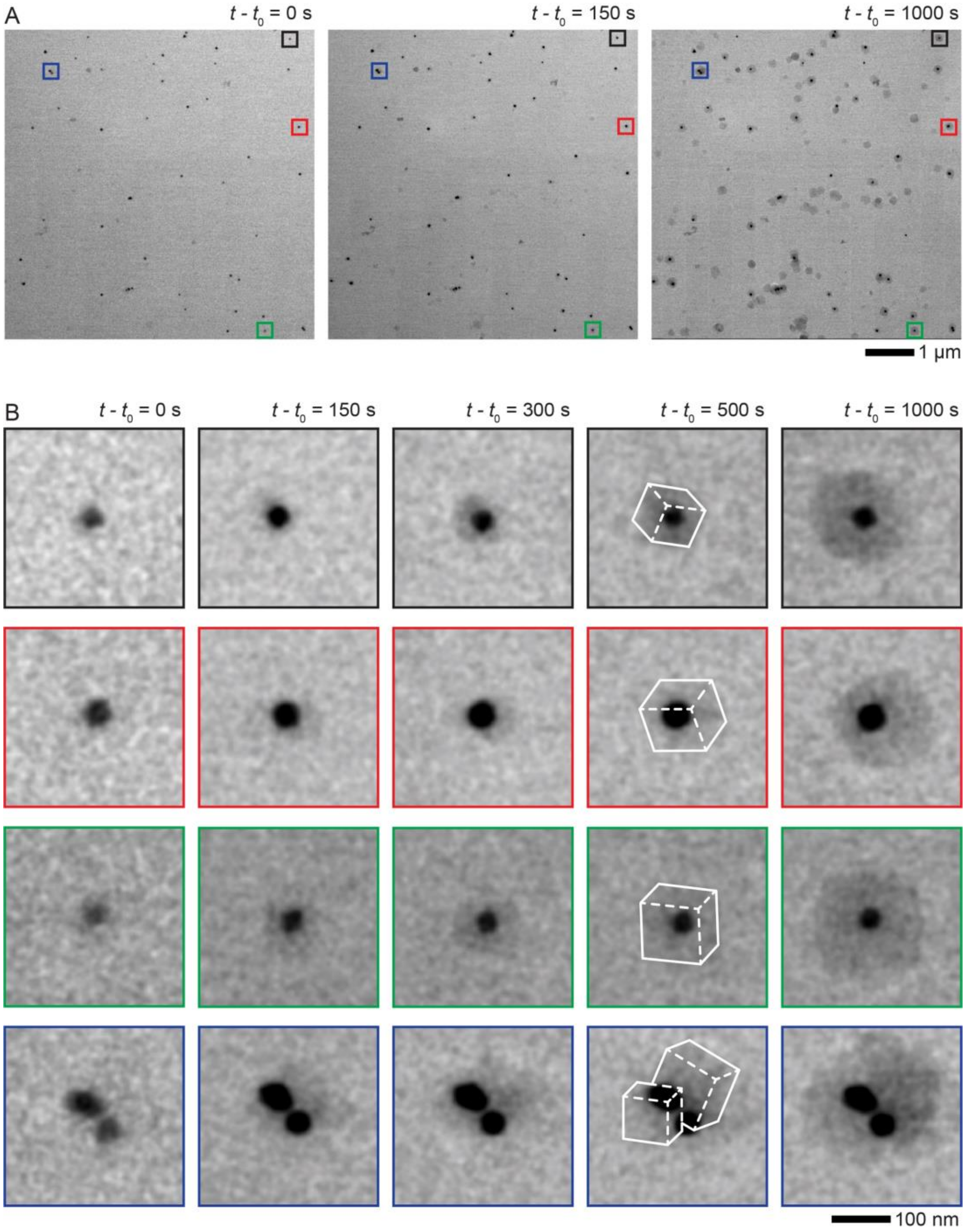

Figure S4. Formation of core−shell Au@ZIF-8 nanostructures in a low-concentration ZIF-8 precursor solution. (**A**) *In situ* TEM image series showing the encapsulation of Au NPs by single or multiple ZIF-8 shells in an aqueous solution comprising 0.4 M 2-MeIm, 6 mM $Zn(NO_3)_2$, and 0.25 mM CTAC in a liquid cell at room temperature (Supporting Video 4). Here, t_0 represents the time point when the imaging started. (**B**) Enlarged views of the TEM image series shown in (A) depicting the details of the formation of four Au@ZIF-8 nanostructures.

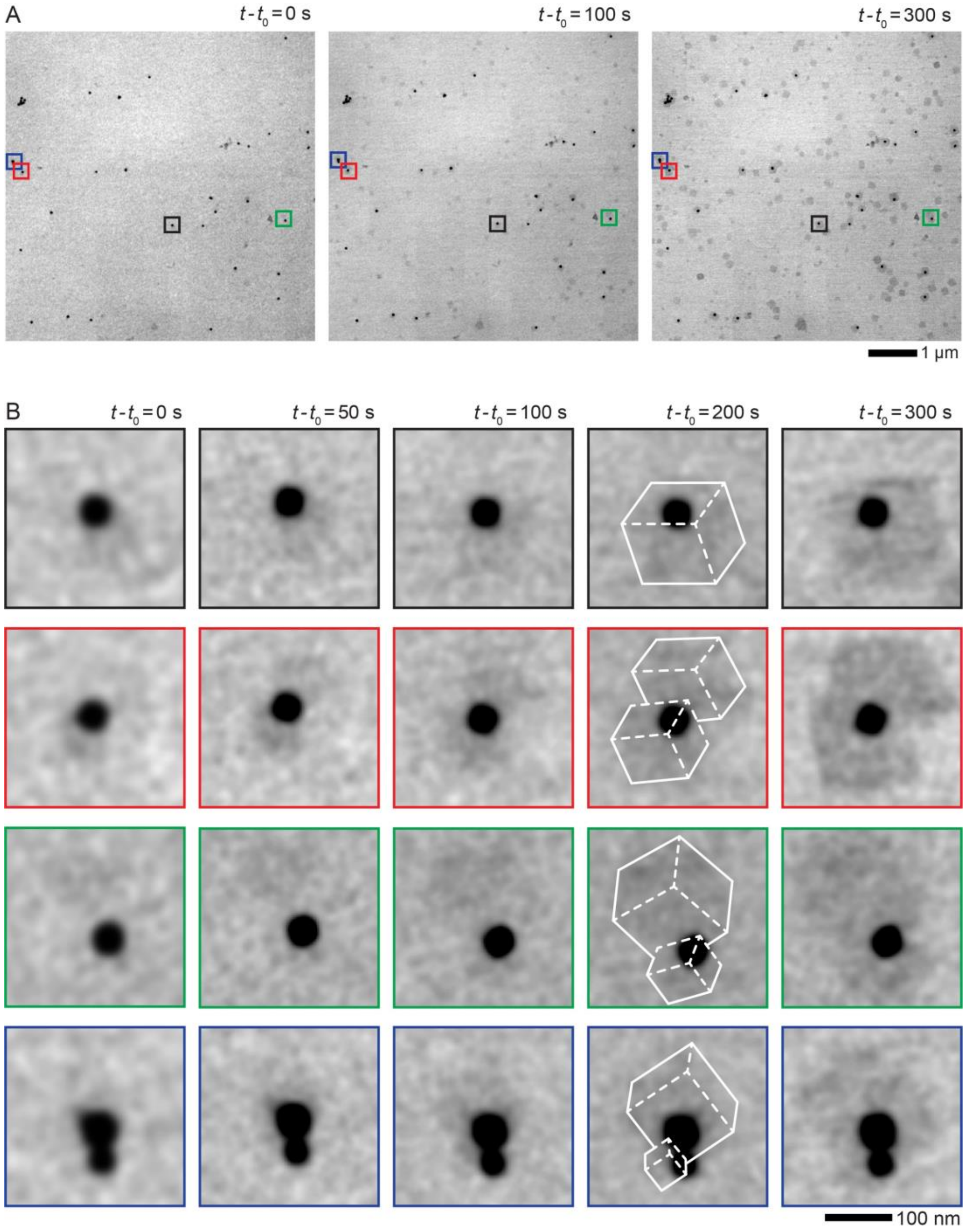

Figure S5. Formation of core−shell Au@ZIF-8 nanostructures in a high-concentration ZIF-8 precursor solution. (**A**) *In situ* TEM image series showing the encapsulation of Au NPs by single or multiple ZIF-8 shells in an aqueous solution comprising 0.6 M 2-MeIm, 9 mM $Zn(NO_3)_2$, and 0.25 mM CTAC in a liquid cell at room temperature (Supporting Video 5). Here, t_0 represents the time point when the imaging started. (**B**) Enlarged views of the TEM image series shown in (A) depicting the details of the formation of four Au@ZIF-8 nanostructures.

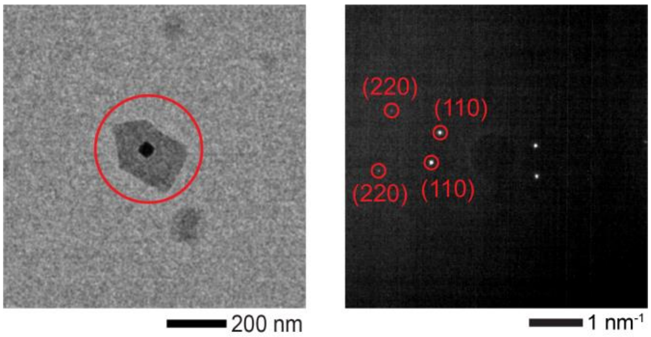

Figure S6. Au@ZIF-8 nanostructures. TEM and electron diffraction images of *in situ* synthesized Au@ZIF-8 nanostructures with a polycrystalline cuboidal ZIF-8 shell obtained from a high-concentration ZIF-8 precursor solution (0.6 M 2-MeIm + 9 mM $Zn(NO_3)_2$ + 0.25 mM CTAC).

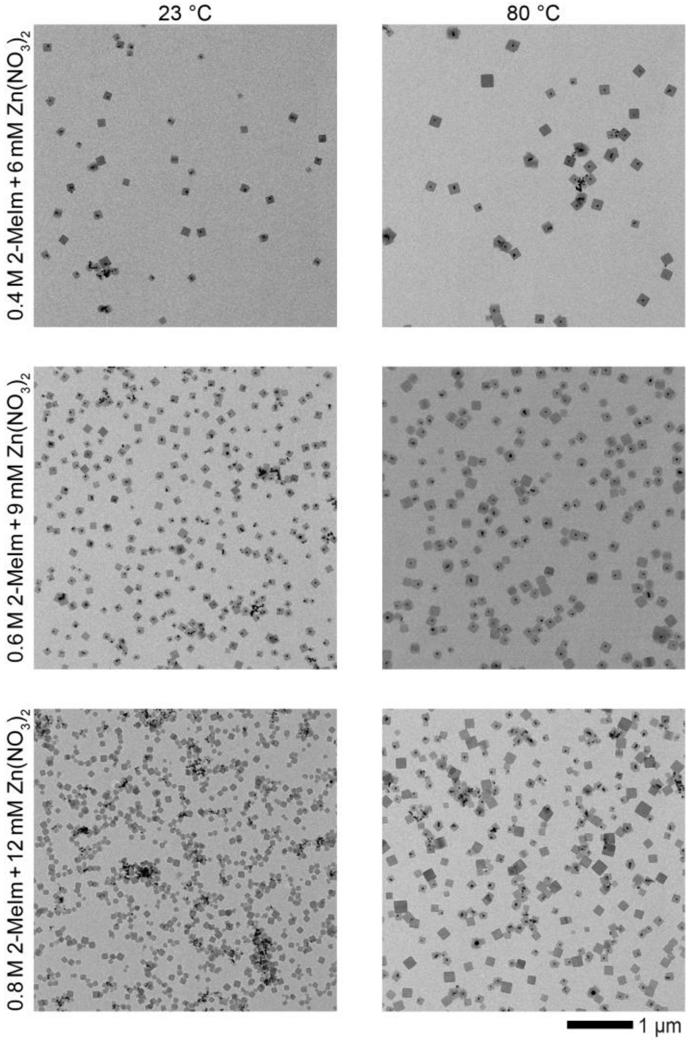

Figure S7. Au@ZIF-8 nanostructures synthesized at different ZIF-8 precursor concentrations and different temperatures. TEM images of Au@ZIF-8 nanostructures synthesized at 23 and 80 °C using the following ZIF-8 precursor solutions: 0.4 M 2-MeIm + 6 mM $Zn(NO_3)_2$ + 0.25 mM CTAC, 0.6 M 2-MeIm + 9 mM $Zn(NO_3)_2$ + 0.25 mM CTAC, and 0.8 M 2-MeIm + 12 mM $Zn(NO_3)_2$ + 0.25 mM CTAC.

2. Supporting video captions

Supporting Video 1: Effect of 200-keV electron beam of flux ~5 e^{-} nm^{-2} s^{-1} on a ZIF-8 shell of pre-synthesized Au@ZIF-8 nanostructures when imaged in a precursor solution comprising 0.6 M 2-MeIm, 9 mM $Zn(NO_3)_2$, and 0.25 mM CTAC as shown in Figure S3A.

Supporting Video 2: Effect of 200-keV electron beam of flux ~0.75 e^{-} nm^{-2} s^{-1} on a ZIF-8 shell of pre-synthesized Au@ZIF-8 nanostructures when imaged in a precursor solution comprising 0.6 M 2-MeIm, 9 mM $Zn(NO_3)_2$, and 0.25 mM CTAC as shown in Figure S3B.

Supporting Video 3: Effect of 200-keV electron beam of flux ~0.5 e^{-} nm^{-2} s^{-1} on a ZIF-8 shell of pre-synthesized Au@ZIF-8 nanostructures when imaged in a precursor solution comprising 0.6 M 2-MeIm, 9 mM $Zn(NO_3)_2$, and 0.25 mM CTAC as shown in Figure S3C.

Supporting Video 4: The *in situ* TEM movie showing the growth of a ZIF-8 shell on Au NPs in a precursor solution comprising 0.4 M 2-MeIm, 6 mM $Zn(NO_3)_2$, and 0.25 mM CTAC as shown in Figures 2A and S4A. The image series were acquired at an electron flux of ~0.5 e^{-} nm^{-2} s^{-1}.

Supporting Video 5: The *in situ* TEM movie showing the growth of a ZIF-8 shell on Au NPs in a precursor solution comprising 0.6 M 2-MeIm, 9 mM $Zn(NO_3)_2$, and 0.25 mM CTAC as shown in Figures 3A, 4A and S5A. The image series were acquired at an electron flux of ~0.5 e^{-} nm^{-2} s^{-1}.
